# Skin-touch-actuated textile-based triboelectric nanogenerator with black phosphorus for durable biomechanical energy harvesting

**DOI:** 10.1038/s41467-018-06759-0

**Published:** 2018-10-15

**Authors:** Jiaqing Xiong, Peng Cui, Xiaoliang Chen, Jiangxin Wang, Kaushik Parida, Meng-Fang Lin, Pooi See Lee

**Affiliations:** 1 School of Materials Science and Engineering, Nanyang Technological University, Singapore, 639798 Singapore; 2 State Key Laboratory for Manufacturing Systems Engineering, Xi’an Jiaotong University, 710049 Xi’an, Shaanxi China; 3 Present Address: School of Aerospace, Transport and Manufacturing, Cranfield University, Cranfield, MK43 0AL UK

## Abstract

Textiles that are capable of harvesting biomechanical energy via triboelectric effects are of interest for self-powered wearable electronics. Fabrication of conformable and durable textiles with high triboelectric outputs remains challenging. Here we propose a washable skin-touch-actuated textile-based triboelectric nanogenerator for harvesting mechanical energy from both voluntary and involuntary body motions. Black phosphorus encapsulated with hydrophobic cellulose oleoyl ester nanoparticles serves as a synergetic electron-trapping coating, rendering a textile nanogenerator with long-term reliability and high triboelectricity regardless of various extreme deformations, severe washing, and extended environmental exposure. Considerably high output (~250–880 V, ~0.48–1.1 µA cm^−2^) can be attained upon touching by hand with a small force (~5 N) and low frequency (~4 Hz), which can power light-emitting diodes and a digital watch. This conformable all-textile-nanogenerator is incorporable onto cloths/skin to capture the low output of 60 V from subtle involuntary friction with skin, well suited for users’ motion or daily operations.

## Abstract (web summary)

Incorporation of triboelectric nanogenerators into textiles is attractive for self-powered wearable electronics. Here the authors employ black phosphorus with a hydrophobic coating in a durable, washable, and air permeable textile-based device that converts biomechanical motion into electricity.

## Introduction

Wearable electronics are rapidly emerging in modern living to meet the requirements of smart fabrics, motion tracking, health monitoring, and wearable light-emitting diodes (LEDs)^1–6^. Electronic modules with desired deformability that can provide maximum freedom are considered as the next-generation wearable electronics^7,8^. Considering the design aspects on practicality and esthetics, these wearable devices are required to be small, lightweight, flexible, and washable; of particular concern is their power units that typically account for a large volume and determine the performance of devices^9^. Tremendous efforts have been dedicated to develop the devices with low power requirements that could be powered with energy storage units such as batteries. However, these rigid batteries require frequent charging or have to be replaced/disposed due to the lack of self-charging capability^10^. Energy-harvesting technologies from the ambient environment are of importance in addressing these issues to ensure the sustainable operation of wearable devices^11^. Since human activities are based primarily on mechanical movement, regardless of climatic conditions and working environment, harvesting the ubiquitous and constantly available biomechanical energy is the most likely reliable and independent strategy for providing a continuous power.

Triboelectric nanogenerators (TENGs) generate electricity by triboelectrification and electrostatic induction from ambient mechanical motion, such as mechanical friction/vibration, rotary motion, oscillating motion and expanding/contracting motion^12^. Various self-charging power systems based on TENGs have been developed owing to merits of light weight, small size, high efficiency, and a wide choice of materials^13–15^. Biomimetic devices inspired by human, animal, and insect skins, such as electronic skin (e-skin), optoelectronic system, skin-like TENG and capacitor^16^, have shown potential applications in prosthetic/robotic skins^16,17^, health monitoring^17,18^, human-interactive interfaces^19^, and military camouflage^20^. A skin-like TENG is a promising module that enables realization of self-powered wearable electronics. Recently, scientists have developed a durable and resilient TENG that mimics the skin of an electric eel and is composed of silicone rubber and a silver nanowires electrode that generates stable electricity from touch under different extreme deformations. A skin-driven working process makes this TENG adaptable for self-powered systems. However, silicone rubber is far from an ideal substrate for long-term wearable electronics due to poor air permeability.

In comparison, textile is a more promising candidate for realizing comfortable wearable electronics since excellent deformability and breathability make it compatible for incorporation with our daily clothes^21–29^. However, these wearable generators are constructed by a dual-electrodes mode that operates based on the friction of adjacent fibers/yarns or face-to-face fabrics with coating of different synthetic polymers or metallic patterns that often lead to poor breathability and low comfort. Although the designs enable sensitivity even for subtle motions, the increased contact and friction between the fibers/yarns impart wear and tear, especially with strongly repetitive mechanical pulses, humidity or light exposed environments, leading to some important concerns about the degradation and lifetime of TENGs.

Textiles are an ideal substrate, but achieving a durable textile-based TENG (textile-TENG) with high performance for harvesting energy from both voluntary and involuntary body motions with different frequency remains challenging. These problems and requirements motivate searches for new materials, particularly the rapid development of two-dimensional (2D) materials has opened new directions. Kim et al. have demonstrated electrical energy harvesting from monolayer and few-layer graphene by mechanical stressing^30^. Recently, Kim et al. further showed that reduced graphene oxide (RGO) and molybdenum disulfide (MoS_2_) serve as efficient triboelectric electron–acceptor layers in negative friction materials for enhancing the electricity output of TENGs, which is attributed to effective charge capture in the RGO or MoS_2_ layer, suppressing the loss of generated triboelectric electrons^31,32^. Thereafter, metallic 2D MXenes (e.g., titanium carbide Ti_3_C_2_T_x_) were confirmed as effective triboelectric materials that are even more negative than Teflon^33^. Recently, Thundat et al. have demonstrated the direct harvesting of large triboelectric direct current using nanoscale sliding friction of a C-AFM tip on a pulsed laser-deposited MoS_2_ thin film, which further reveals the promising application of 2D materials for triboelectricity^34^. Black phosphorus (BP) is an emerging 2D semiconducting material with in-plane anisotropic transport, a tunable direct bandgap and high carrier mobility, making it attractive for applications in optoelectronic devices^35^. BP nanosheets, with high specific surface area and quantum confinement effects, possibly enable the realization of electron accepting properties^36^. However, applications of BP are challenged by a fast degradation on exposure to ambient conditions, especially for the few-layer BP^37^.

To develop a durable skin-touch-triggered textile-TENG with high performance, here we propose a synergetic triboelectric trapping layer of BP with protection by cellulose-derived hydrophobic nanoparticles (HCOENPs) to alleviate degradation. The robust layer is realized on the knitted polyethylene terephthalate (PET) textile by using dip-coating, a facile way to achieve uniform attachment of nanomaterials, meeting the compatibility for large-scale manufacturing. As the charge induced component, the resultant HCOENPs/BP/PET fabric (HBP-fabric) is combined with a fabric electrode and a waterproof fabric to construct our sandwich textile-TENG. It shows excellent water repellency and durability upon washing. An all-fabric-based configuration renders a whole device that can be voluntarily crumpled, twisted and stretched. While applying a force by human skin contact, the textile-TENG can generate electricity via a triboelectric effect to harvest the biomechanical energy. Owing to the electron-trapping capacity of HCOENPs/BP, an instantaneously maximum output voltage and current density of 880 V and 1.1 µA cm^−2^ (peak-to-peak values) can be achieved and is demonstrated to easily power up over 150 LEDs in series. Also, it can generate effective power with loading of varying external resistances. A digital watch is driven by the textile-TENG, promising an attractive concept for deriving a wide range of wearable/deformable electronics and self-powered textiles/human-interactive systems.

## Results

### Fabrication of textile triboelectric nanogenerator

**Fig. 1:**
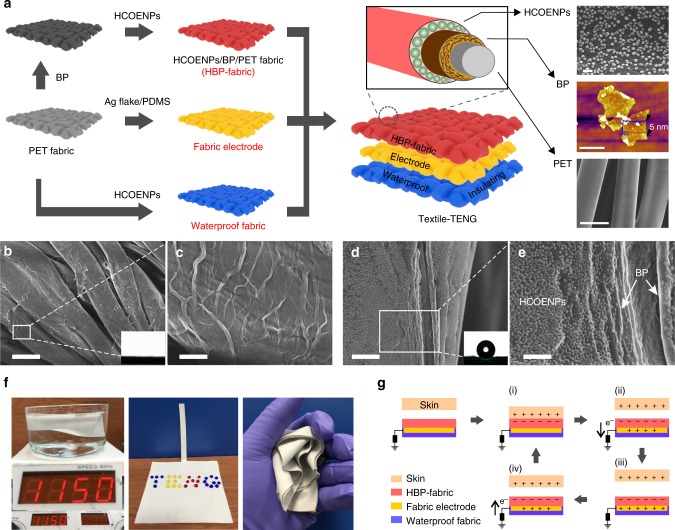
Configuration and working mechanism of skin-touch-actuated textile triboelectric nanogenerator. **a** Schematic illustration of the fabrication process of textile triboelectric nanogenerator (textile-TENG) based on the polyethylene terephthalate (PET) fabrics. The charge-induced component, HCOENPs/BP/PET fabric (HBP-fabric) was realized on a PET fabric, with successive coating of black phosphorus (BP) and hydrophobic cellulose oleoyl ester nanoparticles (HCOENPs). The fabric electrode is a PET fabric enabled by coating with a conductive paste that composed of silver flakes and polydimethylsiloxane (Ag flake/PDMS). PET fabric with coating of HCOENPs layer serves as the waterproof fabric for encapsulating the fabric electrode. Scanning electron microscopy (SEM) and atomic force microscopy (AFM) images on the right side of (**a**) indicate the morphologies of three components of HBP-fabric. HCOENPs, scale bar, 100 nm. BP, scale bar, 2 µm. PET fabric, scale bar, 20 µm. **b** SEM planar top view of continuous BP coating on the PET fibers. Scale bar, 20 µm. **c** Enlarged view of the region marked with a box in **b**, showing the winkled BP sheet on PET fibers. Scale bar, 2 µm. **d** SEM image of a representative HBP-fiber. Scale bar, 1 µm. **e** Enlarged view of the region marked with a box in **d**, revealing the BP layer with dense coating of HCOENPs. Scale bar 300 nm. **f** Photographs demonstrating that textile-TENG is washable and deformable. **g** Schematic illustration of the working mechanism of textile-TENG

Figure 1a depicts a single electrode mode textile-TENG that was composed of three different functional fabrics based on the PET fabric. The foremost triboelectric fabric (HBP-fabric) was realized on PET fabric with successive coating of BP and hydrophobic cellulose oleoyl ester nanoparticles (HCOENPs), which was designed as a durable triboelectric electron–acceptor layer to synergistically enhance the electricity output of TENG. As shown in Fig. 1b, c, liquid-phase exfoliated BP nanosheets (thickness, 5 ± 1 nm, Fig. 1a) were tightly attached to the fiber surface to form a continuous wrinkled coating. Energy-dispersive X-ray spectroscopy (EDS) elemental mapping of the HBP-fabric also confirms a full and uniform coverage (Supplementary Fig. 1). To prevent the fast degradation of BP on exposure to ambient conditions, HCOENPs were prepared (see Supplementary Fig. 2 and Supplementary Note 1) to encapsulate the BP as a continuous dense layer (Fig. 1d, e) in order to protect it against moisture which is vital for accelerated failure of BP^38^. Supplementary Figure 3 presents the surface feature of a HCOENPs-covered-BP on a silicon wafer, demonstrating that HCOENPs can fully encapsulate the BP layer to produce a densely rough surface. The static contact angle of 153° (inset of Fig. 1d) indicates the HBP-fabric is superhydrophobic due to the excellent waterproof performance of HCOENPs layer. To achieve deformability and comfort, the TENG electrode was fabricated by PET fabric with conductive coating which is comprised of silver flakes and polydimethylsiloxane (Ag flake/ PDMS). As shown in Supplementary Fig. 4a–c, Ag flakes with PDMS binding are fully penetrated in the yarns as an interconnected percolating network, rendering a deformable fabric conductive electrode to effectively accommodate the stretchability of textile-TENG. The fabric electrode sustained 600 stretch cycles with stable resistance and morphology, which confirms its good reliability (see Supplementary Fig. 4d). In addition, a waterproof PET fabric can be obtained with the dip-coated HCOENPs and it was used to encapsulate the fabric electrode, forming a sandwich textile-TENG that delivers excellent washability and deformability (Fig. 1f). The device shows a comparable air permeability of 1068 ± 3 L m^−2^ s^−1^ to cotton (1246 ± 3 L m^−2^ s^−1^), the good breathability is due to the all-fabric-based configuration.

### Working mechanism and electrical properties

**Fig. 2:**
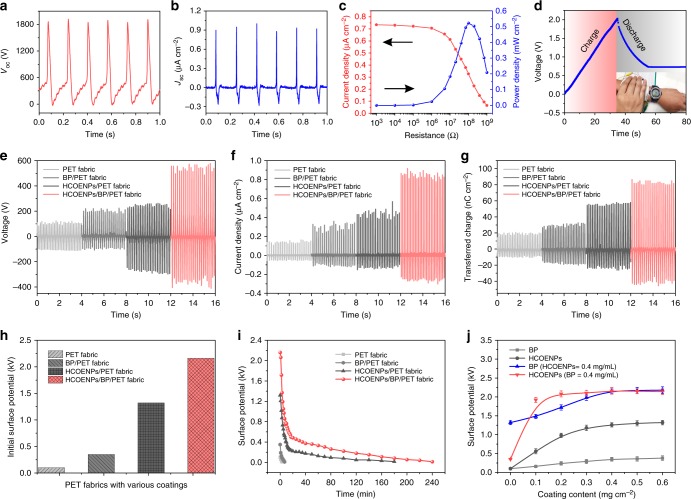
Triboelectric performances of nanogenerators based on modified polyethylene terephthalate fabrics. **a**, **b** Open-circuit voltage (*V*_oc_) and short-circuit current density (*J*_sc_) generated by textile-triboelectric nanogenerator (TENG). **c** Dependence of current density and instantaneous power density of textile-TENG on the resistance of external load. **d** Textile-TENG (7 cm × 7 cm) harvest mechanical energy of hand motion (<5 N, 4 Hz) to charge a 22 µF capacitor for driving a digital watch. **e** Output voltages, **f** current densities, and **g** transferred charges of textile-TENGs based on the bare polyethylene terephthalate (PET) fabric, black phosphorus (BP)-coated PET fabric (BP/PET fabric), hydrophobic cellulose oleoyl ester nanoparticles (HCOENPs)-coated PET fabric (HCOENPs/PET fabric), and HBP-fabric (HCOENPs/BP/PET fabric). 100 MΩ was used as load resistance for output voltage and transferred charge measurement. Operation was performed here by touch force of 5 N and touch frequency of 6 Hz. **h** The initially saturated surface potential of the four different PET-based fabrics. **i** The retention time of the surface potential of four fabrics after friction formed by touching. **j** Dependence of the fabric surface potential on the density of different coatings. Surface potentials are tested here by touching with 5 N and 6 Hz to ensure sufficient induced charges. Error bars represent twice the standard deviations (s.d.)

Textile-TENG, operating with single-electrode mode can be lightly actuated by skin touch, HBP-fabric is the charge induced layer for generating electricity (Fig. 1a). Specifically, the working mechanism is schematically illustrated on the combination of contact triboelectrification and electrostatic induction as shown in Fig. 1g. When the skin contacts with HBP-fabric, charges would transfer from skin to HBP-fabric due to a higher surface electron affinity of fabric (Fig. 1g)^39–42^. Once a relative separation occurs between skin and HBP-fabric, the negative charges on the surface of HBP-fabric will induce positive charges on the fabric electrode to compensate the triboelectric charges, driving free electrons to flow from the fabric electrode to the ground (Fig. 1g). This electrostatic induction process can generate an output voltage/current signal. When the negative triboelectric charges on the HBP-fabric are completely balanced by the induced positive charges on the fabric electrode, no output signals were produced (Fig. 1g). When skin approaches HBP-fabric, the induced positive charges on the fabric electrode decrease, causing the electrons flow from the ground to the fabric electrode until skin and HBP-fabric becomes fully contact with each other again, resulting in a reversed output signal (Fig. 1g). This is a full cycle of the electricity generation process for the textile-TENG. With periodical touch on the HBP-fabric, an alternating electricity output can be continuously generated. Owing to the synergetic enhancement of electron–acceptor of HCOENPs/BP, the measured open-circuit voltage and short-circuit current density can achieve ~1860 V and 1.1 µA cm^−2^ during fast contact/separation between skin and HBP-fabric (Fig. 2a, b).

For different practical applications, the effective output power of textile-TENG was evaluated and calculated as *I*^2^*R/A* via measuring its output current density by externally connecting various resistance loads in series, where *I* is the output current across the external load, *R* is the load resistance, and *A* is the effective contact area. Figure 2c shows the dependence of output power density on the resistances. The current density decreases from 0.74 to 0.07 µA cm^−2^ as the load resistance increases from 0.001 MΩ to 1 GΩ. The maximum instantaneous power density of 0.52 mW cm^−2^ could be achieved when the load resistance is around 100 MΩ. With such high performance, textile-TENG can instantaneously light up over 150 LEDs in series by gentle touch (<5 N, 4 Hz) (Supplementary Fig. 5a). It can charge a capacitor for driving the digital watch (Fig. 2d), showing a much higher power compared with the bare PET fabric that can barely drive about 10 LEDs (see Supplementary Fig. 5b for the comparison).

Interestingly, the HCOENPs layer of the HBP-fabric also serves to enhance the charge induced capacity besides protecting the BP, which is due to its rougher surface (RMS roughness, 21.1 ± 5.4 nm) with efficient triboelectric effect (see Supplementary Fig. 6 for the roughness changes). Therefore, as successive coating of PET fabric, BP and HCOENPs were studied for their respective and concomitant capacities on the triboelectric outputs of fabric, the output voltage and transferred charge were measured with load resistance of 100 MΩ. Figure 2e, f show the outputs of TENGs based on different fabrics with hand touching of 5 N (6 Hz). The device based on pristine PET fabric can generate voltage and current density of 200 V and 0.18 µA cm^−2^, respectively. It is reasonable that PET is an intrinsically negative triboelectric material, which will be charged by inducing with a positive material (such as skin)^12^. After coating with BP, the output voltage and current density increase to 300 V and 0.4 µA cm^−2^, respectively. It should be attributed to the electron-trapping ability of BP, which provides a charge storage layer to suppress the loss of triboelectric electrons for increasing the electricity outputs. With coating of HCOENPs only, expected high outputs of 530 V and 0.6 µA cm^−2^ are attained. Interestingly, the output further increases to 880 V and 1.1 µA cm^−2^ with successive coating of BP and HCOENPs. As presented in Fig. 2g, a similar ascending tendency of their transferred charge densities (from 26 to 125 nC cm^−2^) can be measured, suggesting that there is a synergistic effect of BP and HCOENPs for capturing the induced charges to promote the corresponding outputs. There are significant improvements of outputs with coating of BP or HCOENPs compared with bare PET fabric, which means both BP or HCOENPs can contribute to the density of induced charge. Thus the hypothesized synergistic enhanced effect could be attributed to the BP layer transitorily maintains the induced charges generated from the top layer of HCOENPs, suppressing the charge loss to ensure a more complete transfer process of induced charges between fabric electrode and ground.

To confirm this hypothesis, surface potentials of the fabrics are measured as direct evidences to reveal the respective contribution of BP and HCOENPs. The measurement was performed on fabrics with friction by hand touching. As shown in Fig. 2h, initial surface potentials of BP/PET fabric, HCOENPs/PET fabric, and HCOENPs/BP/PET fabric are 0.35, 1.32, and 2.16 kV, respectively, which can be maintained for longer time than the bare PET fabric (0.1 kV). Especially the fabrics with coating of HCOENPs or BP/HCOENPs project a lasting surface potential to 3 and 4 h after sufficient friction (Fig. 2i). The results of the surface potential test demonstrate that both BP and HCOENPs can act as electron–acceptor coatings for the PET fabric. Notably, it is explicit that BP and HCOENPs play a synergistic enhancement on the induced and stored charges, generating a higher output performance rather than simply combining the effect of each other. To further clarify the enhanced contribution of BP and HCOENPs, we studied the dependence of surface potential on the coating density of BP and HCOENPs. As shown in Fig. 2j, surface potential of BP/PET fabric increases gradually to 0.36 kV with increasing the BP density from 0 to 0.4 mg cm^−2^, then it reached the saturation. In comparison, the surface potential of HCOENPs/PET fabric can quickly attain a balanced value (1.32 kV) when HCOENPs density increases to 0.2 mg cm^−2^. Moreover, when we increase the density of BP or HCOENPs with fixed density (0.4 mg cm^−2^) of another one, the increase of the fabric surface potential showed a similar rising tendency. It indicates that both BP and HCOENPs play important role to promote the frictional surface charges, 0.4 mg cm^−2^ is enough for them to fully cover the fibers to obtain a fabric with saturated surface triboelectric performance. Especially, 0.2 mg cm^−2^ of HCOENPs can construct a continuous protective coating for BP and contribute a stable output for HBP-fabric.

### Extract energy from voluntary and involuntary body motions

**Fig. 3:**
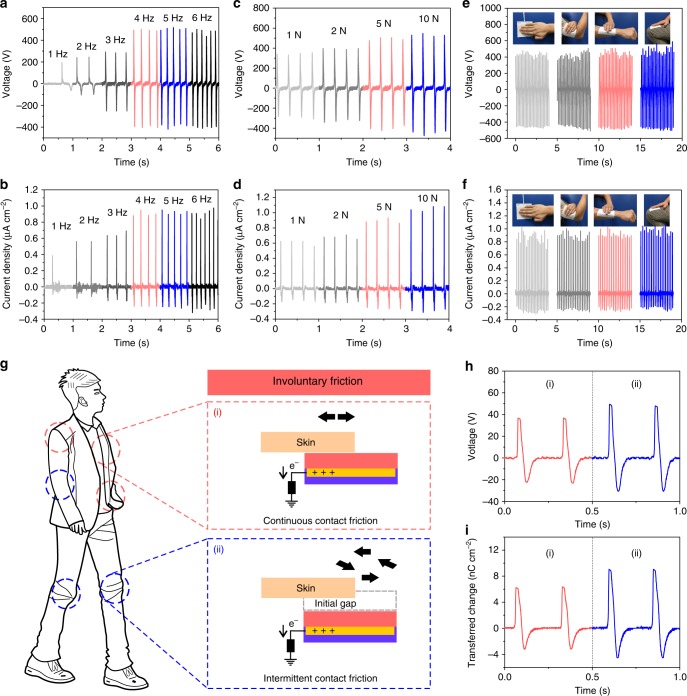
Electrical outputs of textile triboelectric nanogenerator under voluntary and involuntary friction. **a** Output voltages and **b** current densities of textile triboelectric nanogenerator (textile-TENG) under 5 N touch with different frequency. **c** Output voltages and **d** current densities of textile-TENG under 4 Hz touch with different forces. **e** Output voltages and **f** current densities of textile-TENG mounted on different body regions. **g** Schematically illustration of the possible involuntary friction of textile-TENG on different regions of human body. (i) Continuous contact friction (red circles), (ii) Intermittent contact friction (blue circles). The human silhouette image was created by Jiaqing Xiong for use in this figure. **h**, **i** Output voltages and transferred charge densities of textile-TENG in the case of involuntary friction with human body with continuous (red curve, i) or intermittent contact friction (blue curve, ii)

On the basis of the above-mentioned findings, performances of textile-TENG with effective dimension of 7.0 × 7.0 cm^2^ were investigated systematically. Figure 3a, b show its output voltages and current densities by applying a 5 N touching by hand with frequency from 1 to 6 Hz. The generated voltages are 250, 350, 580, 880, 890, and 860 V. The corresponding current densities are 0.48, 0.65, 0.8, 1.1, 1.1, and 1.1 µA cm^−2^ (Fig. 3b). Figure 3c, d depict the output voltages and current densities of textile-TENG under 4 Hz with applied force of 1, 2, 5, and 10 N, which achieve 630, 750, 880, and 1000 V, respectively. The relevant current densities attain 0.78, 0.85, 1.1, and 1.3 µA cm^−2^. These results demonstrate that the outputs depend on the touch frequency more than the touch force, which is reasonable as sufficient charges should be only induced and maintained by an enough sequential touch. Touch of 1 N with 4 Hz is enough to attain a relatively high current density that is superior than the reported micropyramid structured PDMS TENG and the silicone rubber skin-like TENG^38,39^. To confirm that textile-TENG can be utilized on human body to harvest biomechanical energy, we mounted it directly on skin or cloth at different locations of body to study its output performances. As shown in Fig. 3e, f, textile-TENG can fully fit different body regions owing to its excellent deformability, upon touching with 5 N of 5 Hz, it produces stable output voltages and current densities at different body locations.

The aforementioned performances of textile-TENG were measured based on the voluntary touch by hand to systematically evaluate the device. It demonstrates that textile-TENG can generate considerable electricity even under small touch force with low frequency. Thus, it can harvest the low frequency body motion as far as possible, meeting different operation requirements from users. From a practical point of view, a wearable textile TENG should be able to directly contact with skin, thus, involuntary friction will occur between the device and skin that is the same as the friction between clothes and skin. Therefore, our textile-TENG was mounted on body to confirm its high sensitivity for harvesting the small body motions by the involuntary friction (<1 N). Two different frictional processes could take place on different body location (Fig. 3g), either with or without the initial gap or separation between the device and skin, we distinguish them as continuous contact friction ((i), without gap) and intermittent contact friction ((ii), with initial gap), respectively (see Supplementary Fig. 7 for the detailed working processes). Accordingly, the output voltages and transferred charge densities of textile-TENG based on these two friction processes are shown in Fig. 3h, i, respectively. Output voltages of 60 and 80 V can be achieved with relevant transferred charge densities of 9 and 12.8 nC cm^−2^, respectively.  It indicates that our textile-TENG possesses high sensitivity to effectively harvest the biomechanical energy from both the voluntary and involuntary body motions.

### Deformability and durability of device

**Fig. 4:**
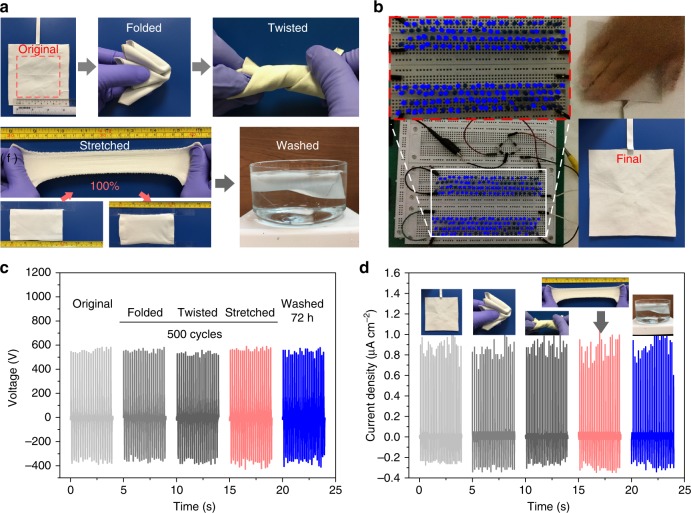
Deformability and electrical stability of textile triboelectric nanogenerator under various extreme conditions. **a** Photographs demonstrating that the textile triboelectric nanogenerator (textile-TENG) possesses excellent endurance for successively experiencing deformations of fold, twist, and stretch, as well as severe washing. **b** Textile-TENG still can light up around 150 light-emitting diodes (LEDs) after extreme treatments. Touch motion (<5 N) with 4 Hz was used for driving the LEDs. **c** Output voltages and **d** current densities of textile-TENG after suffering various extreme deformations and severe washing. Stretching of 50% strain was performed as well for the measurement

Deformability and durability are important attributes of the wearability of devices. Thus, textile-TENG was tested for its capability of generating electricity after underwent various extreme conditions, including deformations and washability. As shown in Fig. 4a, a planar textile-TENG with effective contact area of 7.0 × 7.0 cm^2^ can be willfully folded, twisted, and even stretched 100% with nearly complete elastic resilience. This excellent deformability and stretchability is attributed to the all-fabric construction of textile-TENG. Thereafter, a device that underwent the above extreme deformations was further severely washed for 24 h, after which the device was confirmed to still drive around 150 LEDs by gently touching (<5 N, 4 Hz) (see Fig. 4b, Supplementary Movie 1 and Movie 2), suggesting an admirable reliability of textile-TENG regardless of extreme treated conditions. The device robustness was also proved by testing the outputs of device after subjected to those harsh conditions for multiple cycles. As shown in Fig. 4c, d, the output voltages and current densities are constant with the original outputs after 500 cycles of fold, twist, and stretch, and it even maintains the high performance after severe washing (stirring rate, 1150 rpm) for 72 h. This remarkable durability of textile-TENG is attributed to the tightly attached coating of BP and HCOENPs, especially the robust protection and waterproof properties provided by HCOENPs would render it with resistance against various extreme conditions (see Supplementary Fig. 8 for more details).

**Fig. 5:**
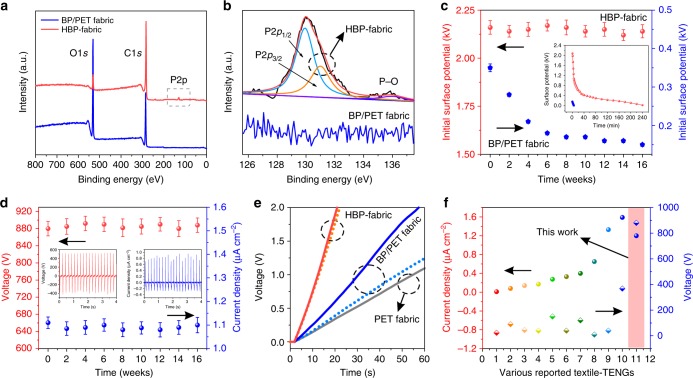
Durability of textile triboelectric nanogenerator under exposure to ambient conditions. **a** X-ray photoelectron spectroscopy (XPS) spectra of BP/PET fabric and HBP-fabric that were stored for 11 weeks. The former is the black phosphorus coated polyethylene terephthalate fabric (BP/PET fabric), the latter (HBP-fabric) is PET fabric with successive coating of BP and hydrophobic cellulose oleoyl ester nanoparticles (HCOENPs). **b** Deconvolution of phosphorus peaks in BP/PET fabric and HBP-fabric, respectively. **c** The initially saturated surface potentials of BP/PET fabric and HBP-fabric within 16 weeks. Inserts are the sustained time of their surface potential after exposure to air for 16 weeks. **d** Periodic monitoring of the peak-to-peak output voltages and current densities of textile-TENG within 16 weeks. Insert are the outputs of device placed in ambient environment after16 weeks. **e** Charging curve of 16 μF capacitor by devices with fabrics of PET, BP/PET, and HBP-fabric. Solid and dashed curves denote the initial charging curves and charging curves after 16 weeks, respectively. **f** Comparison with selected important works on textile TENGs. 1–10 are various TENGs reported based on silver-coated polytetrafluoroethylene (PTFE) textile (TENG-1)^28^, Nylon/PET woven textile (TENG-2)^24^, polydimethylsiloxane (PDMS)-coated stainless steel/PET textile (TENG-3)^26^, reduced graphene oxide (rGO)/Ni coated PET textile (TENG-4)^45^, silicone rubber-coated stainless steel/PET textile (TENG-5)^27^, Kapton/Ni belt woven textile (TENG-6)^46^, polyester core-shell-yarn-based TENG (TENG-7)^47^, Cu/polyimide (PI) wrapped PET textile (TENG-8)^25^, PDMS/Al wire fabric (TENG-9)^21^, Al NPs/PDMS-coated textile (TENG-10)^23^, and this work (TENG-11). Error bars represent twice of the standard deviations (s.d.)

Apparently, BP and HCOENPs without delamination or failure is essential to ensure the reliability of textile-TENG. Especially on the challenge of a fast degradation of BP on exposure to ambient conditions, here, a supposed protection of HCOENPs for the BP was verified by characterizing the HBP-fabric after exposure to air for 11 weeks. Firstly, scanning electron microscopy (SEM) images and element mapping (Supplementary Fig. 9) reveal the fiber was surrounded by an undamaged coating that is composed of BP and HCOENPs. Moreover, we compared the X-ray photoelectron spectroscopy (XPS) spectrum of BP/PET fabric and HBP-fabric to further confirm the state of phosphorus. Deconvolution peaks (Fig. 5a, b) indicate there is a peak of phosphorus presented in HBP-fabric instead of BP/PET fabric, and only a small peak of P-O can be observed at the side of the P 2*p*_1/2_ and P 2*p*_3/2_ peak, demonstrating that BP was protected well on the fibers by HOCENPs coating. Furthermore, the long-term surface potential was monitored (Fig. 5c) with adequate friction by touching, BP/PET fabric shows a markedly decreased surface potential after being exposed to air for 6 weeks. By contrast, HCOENPs/BP/PET fabric maintains a consistent surface potential even after 16 weeks, a potential of 2.08 kV still can be induced with retention of 4 h (insert of Fig. 5c) that is consistent with the initial results (Fig. 2h, i). Therefore, the device outputs show nearly no degradation after exposure in air for 16 weeks (Fig. 5d). Our durable textile-TENG devices were aged in ambient condition (70–80% humidity, 24 ± 3 °C) after 16 weeks and were used to charge a 16 μF capacitor. As shown in Fig. 5e, a decreased charge efficiency was observed from the BP/PET fabric-based device, which is close to that of bare PET fabric device due to the disappearance of BP. In contrast, the textile-TENG based on HBP-fabric maintains an almost identical charge efficiency to that of 16 weeks ago. Thus, it is proved that HCOENPs protected BP is a reliable electron–acceptor coupling  layer for improving the triboelectricity.

Textile-TENG is highly conformal and promising to long-term usage with self-supporting energy. In contrast to other self-powered textile devices that were coated with dense polymer films or designed with complicated dual-electrodes mode, our device delivers an exceptional advantage that it is a high-performance self-powered textile from actuation of human motion (Fig. 5f). Leveraging on the simple and conformable construction of several fabrics, it can be easily incorporated on other textiles or human skin for harvesting biomechanical energy. Also, it is promising to self-powered multifunctional e-fabric or e-skin uses by integrating with other components.

## Discussion

In summary, a skin-touch-actuated wearable triboelectric nanogenerator has been demonstrated on a textile; it is an intrinsically durable and washable textile-TENG with the capability to generate electricity regardless of various extreme conditions. It can harvest the mechanical energy from both voluntary and involuntary body motions, and the maximum instantaneous output electricity can achieve 880 V and 1.1 µA cm^−2^ upon applying voluntary touch with ~5 N and ~4 Hz. Also it can sensitively harvest outputs of 60 V and 9 nC cm^-2^ under involuntary friction with skin. We demonstrate the coupling of BP and HCOENPs as a durable triboelectric electron-trapping coating, enabling an excellent reliability for textile-TENG due to the additional protection of HCOENPs. The all-fabric-based configuration delivers a conformable textile-TENG with extreme deformability and high durability, rendering it with a facile incorporation on clothes or skin and achieving sensitive energy harvesting capacity for body motions of various strength to meet different operation requirements, making it a promising concept for developing self-powered textiles and multifunctional wearable electronics.

## Methods

### Synthesis of hydrophobic coating and BP

Hydrophobic coating of cellulose oleoyl ester nanoparticles (HCOENPs) with sizes of around 35 nm (±5 nm) were synthesized by a nontoxic esterification method and nanoprecipitation technology based on the microcrystalline cellulose^43^. Ethanol suspension of HCOENPs (0.5 mg mL^−1^) was used here (see Supplementary Note 1 for the preparation details). Few layer BP was prepared from the pre-grinded BP by successive sonication (3 h) and centrifugation (10k rpm for 1 h), an ethanol solution of few layer BP (0.5 mg mL^−1^) was used in this work^44^.

### Fabrication of textile triboelectric nanogenerator

PET fabric (thickness, 260 ± 20 µm, dimension, 10 cm × 10 cm) was pretreated by stirring in a 0.2 mol L^−1^ NaOH aqueous solution at 50 °C for 120 min to remove the impurities, followed by rinsing with deionized water to fully wipe off the residual alkali. The resultant dry PET fabric was coated with BP ethanol suspension (0.2 mg mL^−1^) by dip-coating. This is followed by coating with HCOENPs via dip-coating or spray-coating to obtain the HBP-fabric as a triboelectric fabric. Silver flake mixed with PDMS (weight ratio > 2:1) as conducting medium was coated onto the PET fabric via dip-coating to attain the fabric electrode. Another PET fabric was dip-coated with HCOENPs to achieve a waterproof fabric for encapsulating the electrode, making the whole device waterproof and insulating for safe usage. Fabric electrode with dimension of 7 cm × 7 cm was mounted between the HBP-fabric and waterproof fabric with the aid of double-side tape, which was connected externally with a conductive fabric to assemble the textile-TENG for output measurement.

### Characterization and electrical measurement

Atomic force microscope (AFM, Asylum Research, Cypher S) was conducted to evaluate the size of BP nanosheet. Field emission scanning electron microscopy (FE-SEM, JEOL 7600) was employed to reveal the morphologies of BP and HCOENPs on the fibers. EDS and XPS (VG Thermo, Escalab 220i-XL) analysis were performed to confirm the presence of BP. Fourier transform infrared spectrometer (FTIR, PerkinElmer Frontier) and a nanoparticle analyzer (Horiba, SZ-100) were used to determine the structure and size of HCOENPs. Static contact angle of textile was measured by a video-based optical contact angle measuring system (Dataphysics OCA15 Pro) with droplets of 6 µL. Air permeability was tested with a piece of fabric (20 cm^2^) under 100 Pa differential pressure by a fully automatic permeability instrument (YG461E-III). The surface potential of textiles was measured by an Electrostatic Voltmeter (Trek, Model 542 A). The output voltage of TENGs was measured by a mixed domain oscilloscope (Tektronix MDO3024, impedance = 100 MΩ). The output current was measured by a low-noise current preamplifier (Stanford Research System, model SR570, impedance = 4 Ω). The open-circuit voltage (*V*_oc_) and transferred charge were measured by a Keithley 6517B system electrometer (impedance > 200 TΩ). The voluntary body motion was applied by touching with different forces (1–10 N) and frequencies (1–6 Hz) to systematically evaluate the device. The involuntary body motions were achieved by mounting the device on forearm and knee, simulating the real movements of human that include continuous contact friction and intermittent contact friction (initial gap ~0.5 mm). Electrical measurements were carried out with informed signed consents from the human subject.

## Electronic supplementary material

Supplementary Information

Description of Additional Supplementary Files

Supplementary Movie 1

Supplementary Movie 2

## Data Availability

The authors declare that the data supporting the findings of this study are available within the article and its Supplementary Information files. Numerical values of data shown as graphs are available from the corresponding authors upon reasonable request.
