# Supplementary material for: Skin-touch-actuated textile-based triboelectric nanogenerator with black phosphorus for durable biomechanical energy harvesting
Source: Nat Commun. 2018 Oct 15;9:4280. doi: 10.1038/s41467-018-06759-0 (PMC6189134; doi:10.1038/s41467-018-06759-0)
Supplement: Supplementary file 1 — Supplementary Information [file 41467_2018_6759_MOESM1_ESM.pdf]

Supplementary Information

Skin-touch-actuated textile-based triboelectric nanogenerator with black phosphorus for durable biomechanical energy harvesting

Xiong et al.

Supplementary Figures.

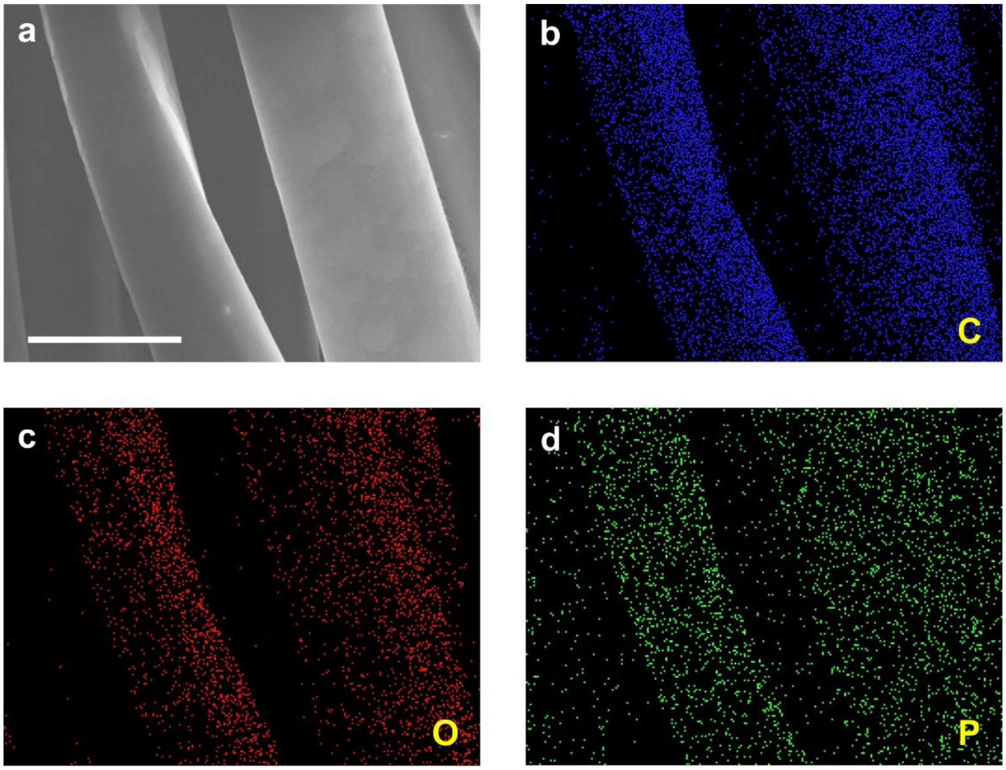

Supplementary Figure 1. Energy-dispersive X-ray Spectroscopy element mapping of HBP-fabric. (**a**) SEM planar view image of fibers in HBP-fabric (HCOENPs/BP/PET fabric), which is a polyethylene terephthalate (PET) textile with successive coating of black phosphorus (BP) and hydrophobic cellulose oleoyl ester nanoparticles (HCOENPs). Scale bar, 20 µm. (**b-d**) The corresponding elemental maps of **a**, demonstrating the full and uniform coverage of BP layer.

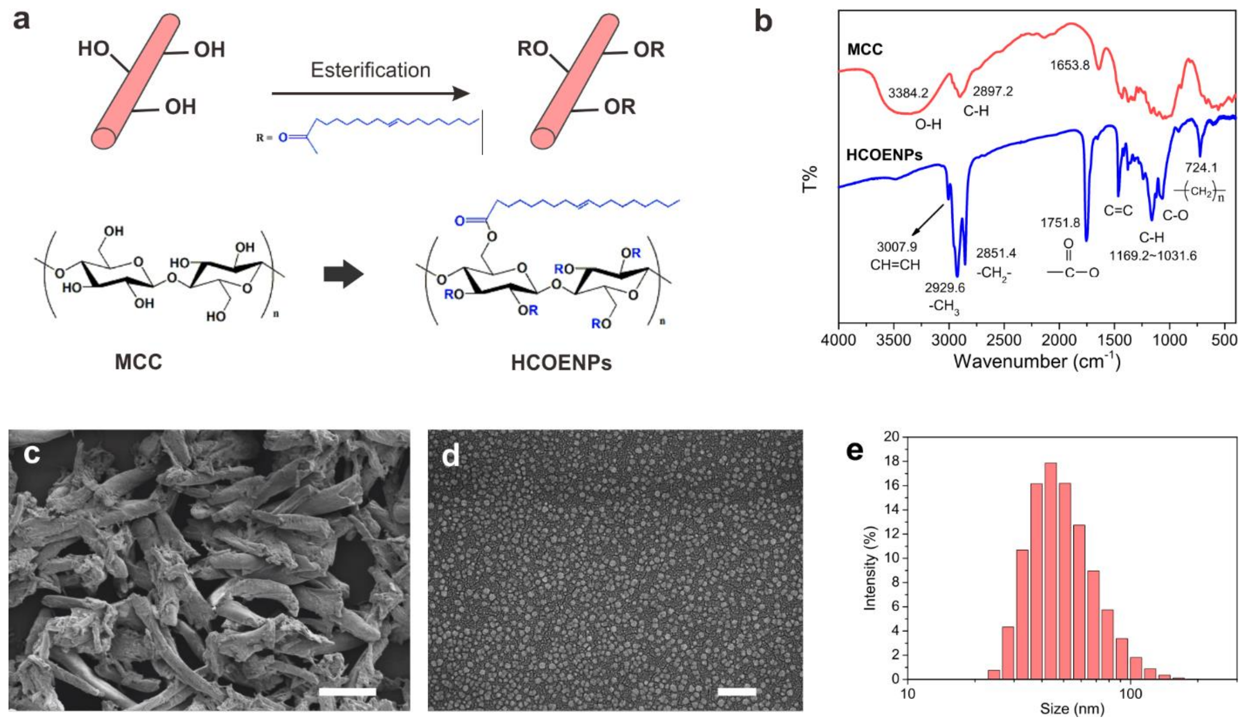

Supplementary Figure 2. Synthesis and characterization of hydrophobic cellulose oleoyl ester nanoparticles. (**a**) Schematic synthesis process of hydrohphobic cellulose oleoyl ester (HCOE). See Supplementary Note 1 for the details. (**b**) FTIR spectra of microcrystalline cellulose (MCC) and hydrophobic cellulose oleoyl ester nanoparticles (HCOENPs) for the demonstration of successful synthesis of HCOE. (**c**) SEM images of MCC. Scale bar, 50 μm. (**d**) HCOENPs with obvious size change compared to MCC. Scale bar, 200 nm**.** (**e**) Size distribution of HCOENPs (Performed by Intensity Mode), demonstrating the HCOENPs possesses uniform size of 50 nm ± 30 nm in the suspension.

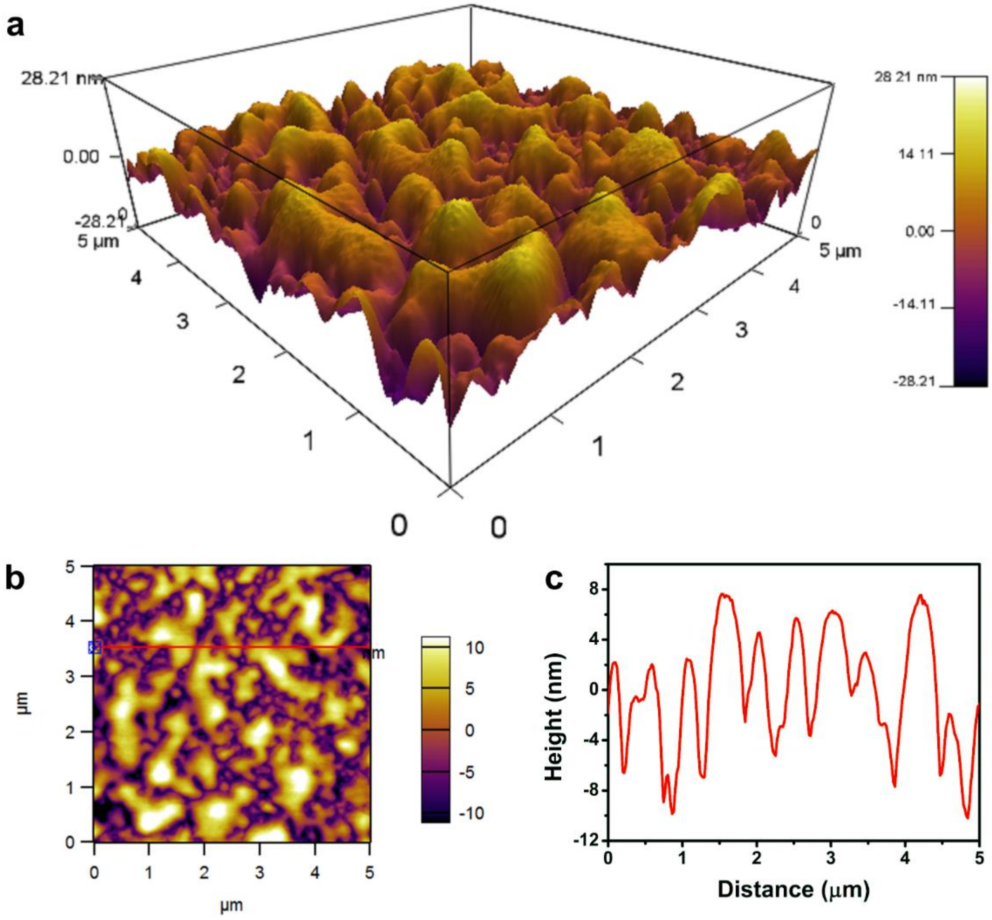

Supplementary Figure 3. Morphology of encapsulated black phosphorus layer on a flat substrate. (**a**) Three dimensional (3D) morphology of a sample that is BP layer with coating of HCOENPs (HCOENPs-coated BP) on silicon wafer. (**b**) Surface topography of the HCOENPs-coated BP. (**c**) Height profiling of the HCOENPs-coated BP.

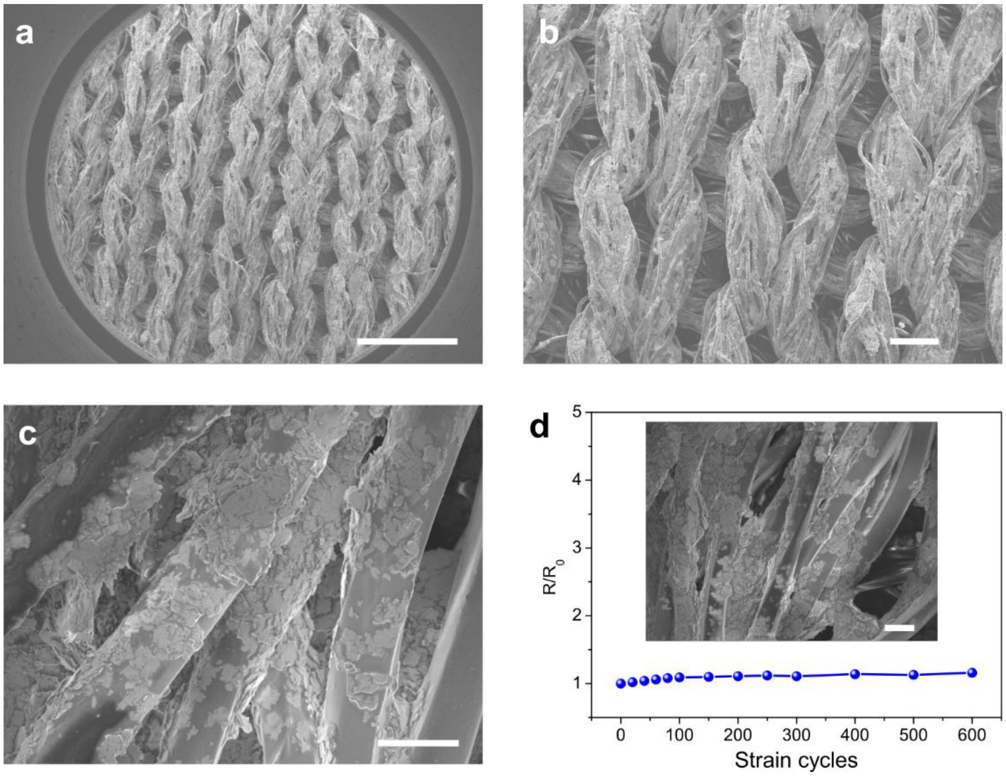

Supplementary Figure 4. Morphology of the fabric electrode. (**a**) Low magnification SEM images of the knitted PET fabric with coating of conductive paste of silver flake and polydimethylsiloxane (Ag flake/PDMS). Scale bar, 1mm. (**b, c**) High magnification SEM images of fabric electrode, demonstrating the Ag flakes are tightly attached on the fibers by binding of PDMS. Scale bar, 200 μm (b), 20 μm (c). (**d**) Stable resistance and unbroken morphology of fabric electrode after 600 cycles with 50% strain. Scale bar of inset, 20 μm.

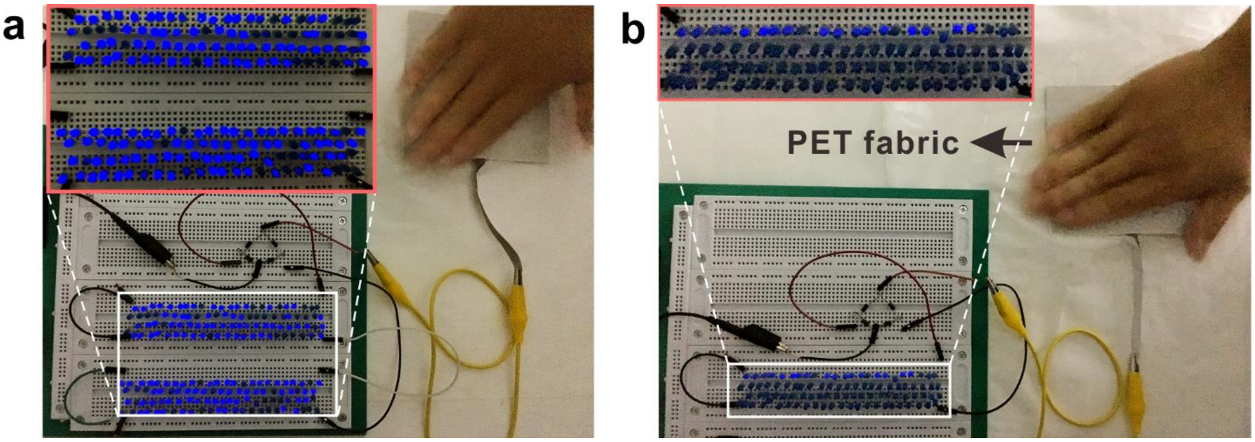

Supplementary Figure 5. Photographs demonstration of biomechanical energy harvested by textile triboelectric nanogenerators for driving the light-emitting diodes. (**a**) A freshly prepared textile triboelectric nanogenerator (textile-TENG) based on HBP-fabric (HCOENPs/BP/PET fabric) can drive around 150 commercial light-emitting diodes (LEDs). (**b**) Device based on a bare PET fabric only can drive about 10 LEDs. Touch force is 5 N, touch frequency is 4 Hz. The effective dimensions of devices operated here is 7 cm × 7 cm. LEDs are connected in series.

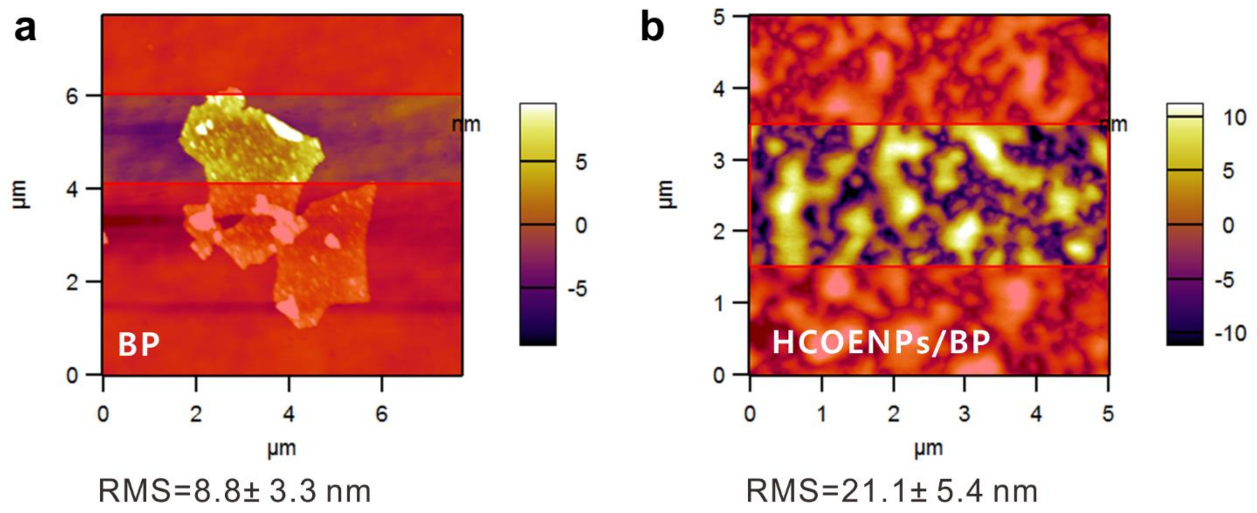

Supplementary Figure 6. Comparison of the surface roughness of black phosphorus before and after coating with hydrophobic cellulose oleoyl ester nanoparticles on a flat substrate. (**a**) AFM image of the BP nanosheets. RMS roughness, 8.8 ± 3.3 nm. (**b**) AFM image of the HCOENPs coated BP layer. RMS roughness, 21.1±5.4 nm.

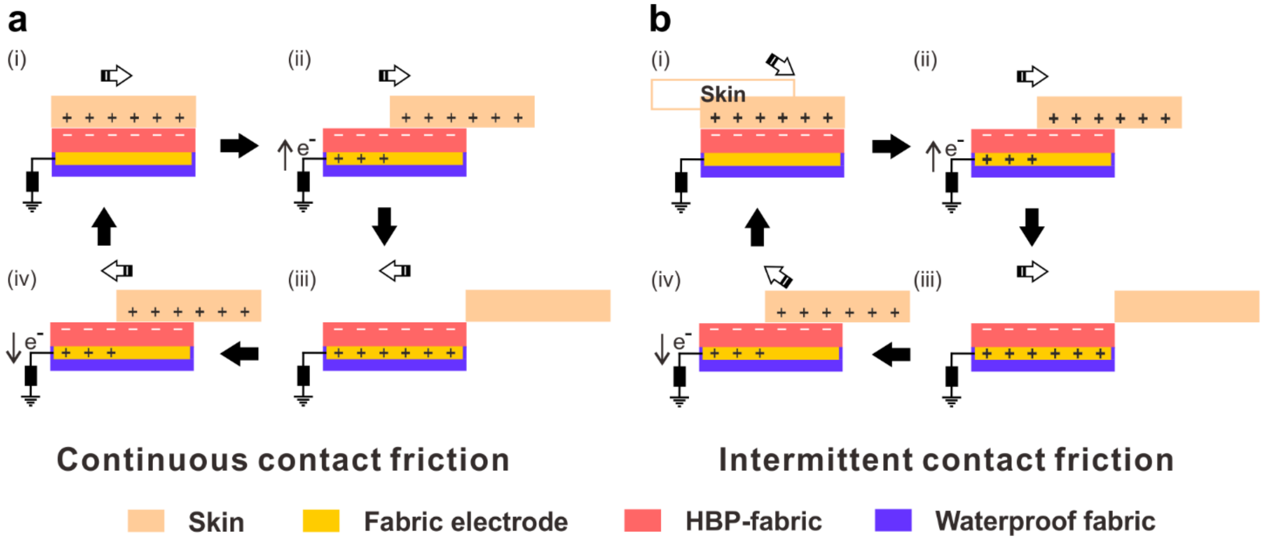

Supplementary Figure 7. The working mechanisms of textile triboelectric nanogenerator under involuntary friction with skin. (**a**) Working process of textile-TENG under involuntarily continuous contact friction. (**b**) Working process of textile-TENG under involuntarily intermittent contact friction.

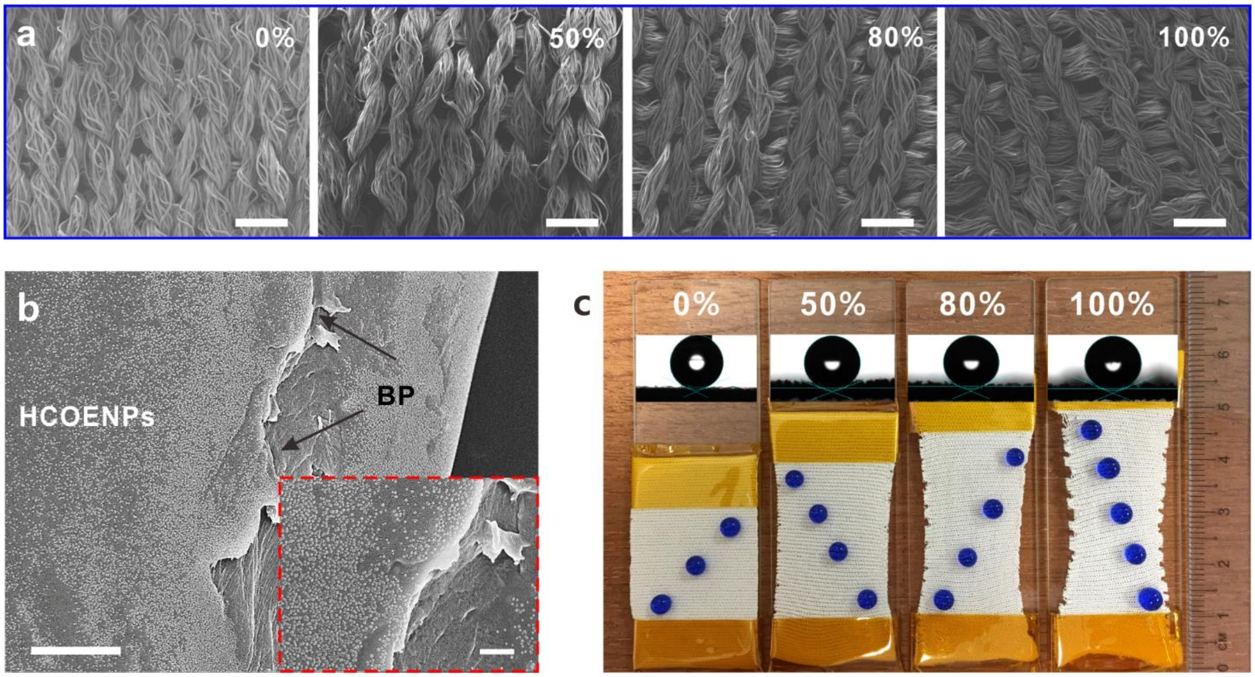

Supplementary Figure 8. Demonstration of stretchability of black phosphorus/ hydrophobic cellulose oleoyl ester nanoparticles coating on the HBP-fabric. (**a**) SEM surface images of HBP-fabric during various stretching, indicating the displacement only occurred between the yarns, scale bar 500 μm. (**b**) High magnification SEM surface image of fibers to reveal the maintained continuous coating of BP/HCOENPs under 100% stretch. Scale bar, 2 μm, scale bar of inset, 400 nm. (**c**) Stable water repellency and static contact angles of the HBP-fabrics under different stretching states, demonstrating the HCOENPs coating for encapsulation is unbroken even under 100% stretch.

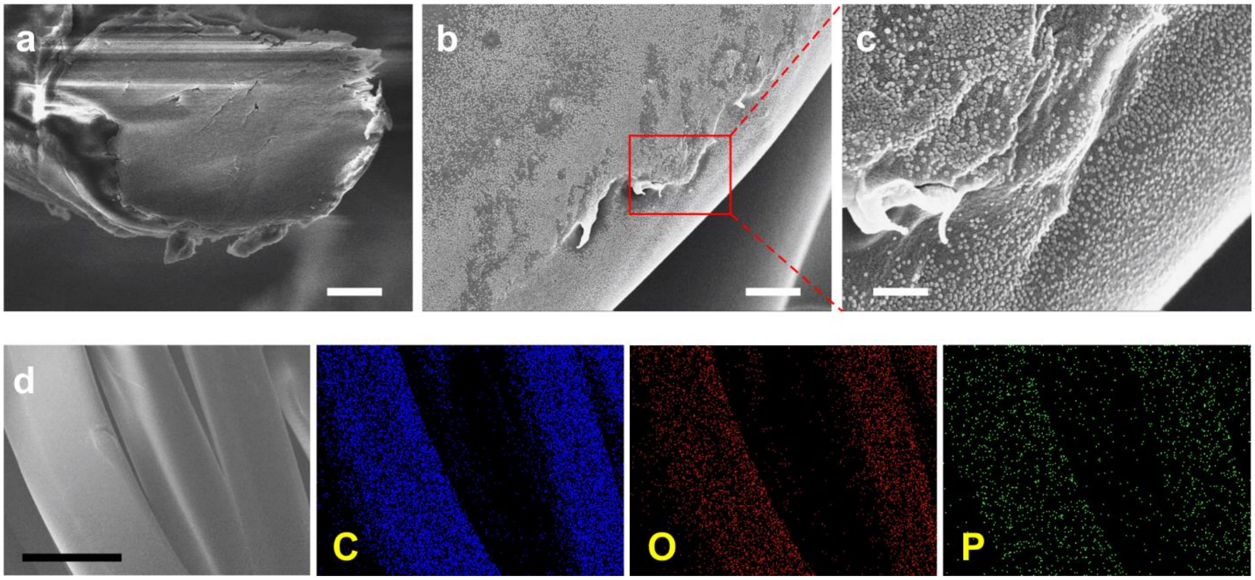

Supplementary Figure 9. Demonstration of the morphologies of HBP-fabric exposed to air for 11 weeks. (**a**) SEM image of the cross section of PET fiber. Scale bar, 2 µm. (**b, c**) SEM images of the PET fibers with successive coating of BP and HCOENPs. Scale bar, 2 µm (b), 400 nm (c). (**d**) SEM planar view image of HBP-fabric and the corresponding elemental maps, demonstrating the uniform distribution of BP on fibers even after exposure to air for 11 weeks. Scale bar, 20 µm.

Supplementary Notes.

Supplementary Note 1. Synthesis of Hydrophobic Cellulose Oleoyl Ester Nanoparticles.

Microcrystalline cellulose, as the starting material was performed with esterification and nanoprecipitation to synthesize the hydrophobic cellulose oleoyl ester nanoparticles (HCOENPs). Typically, 2 g microcrystalline cellulose (MCC, 50 μm, Sigma-Aldrich) was dried thoroughly, suspending in 80 ml pyridine and was heated up to 100℃, while the system was purged with nitrogen, followed by adding 10-28 ml oleoyl chloride, and the reaction process was performed at 100-110 ℃ for 60-90 min. Thereafter, 240-400 ml ethanol/methanol was poured into the mixture to precipitate the product, then which was removed by filtration, and the product was further purified 3-5 times via repeated dissolution in the hexane and precipitation in ethanol/methanol. The resultant cellulose oleoyl ester solution (0.5-5 wt%) was dropwise added into the polar solvent such as ethanol, methanol, isopropanol, etc. under high-speed stirring at room temperature to obtain the suspension of HCOENPs (0.2wt%) with the size about 30±5 nm.
