# Supplementary material for: Skin-touch-actuated textile-based triboelectric nanogenerator with black phosphorus for durable biomechanical energy harvesting
Source: Nat Commun. 2018 Oct 15;9:4280. doi: 10.1038/s41467-018-06759-0 (PMC6189134; doi:10.1038/s41467-018-06759-0)
Supplement: Supplementary file 2 — Description of Additional Supplementary Files [file 41467_2018_6759_MOESM2_ESM.pdf]

Description of Additional Supplementary Files

File Name: Supplementary Movie 1
Description: Demonstration of textile-TENG powering the LEDs after subjected to various extreme mechanical deformations, including stretching (100% strain), twisting, folding and crumpling.

File Name: Supplementary Movie 2
Description: Demonstration of textile-TENG powering the LEDs after subjected to severe washing. Washing was performed by high-speed stirring under room temperature for 24 h.
